# Interplay between Endophyte Prevalence, Effects and Transmission: Insights from a Natural Grass Population

**DOI:** 10.1371/journal.pone.0139919

**Published:** 2015-10-13

**Authors:** Anaïs Gibert, Danièle Magda, Laurent Hazard

**Affiliations:** 1 INRA, UMR AGIR, 31320, Castanet Tolosan, France; 2 Department of Biological Sciences, Macquarie University, Sydney, NSW 2109, Australia; Graz University of Technology (TU Graz), AUSTRIA

## Abstract

Two main mechanisms are thought to affect the prevalence of endophyte-grass symbiosis in host populations: the mode of endophyte transmission, and the fitness differential between symbiotic and non-symbiotic plants. These mechanisms have mostly been studied in synthetic grass populations. If we are to improve our understanding of the ecological and evolutionary dynamics of such symbioses, we now need to determine the combinations of mechanisms actually operating in the wild, in populations shaped by evolutionary history. We used a demographic population modeling approach to identify the mechanisms operating in a natural stand of an intermediate population (i.e. 50% of plants symbiotic) of the native grass *Festuca eskia*. We recorded demographic data in the wild over a period of three years, with manipulation of the soil resources for half the population. We developed two stage-structured matrix population models. The first model concerned either symbiotic or non-symbiotic plants. The second model included both symbiotic and non-symbiotic plants and took endophyte transmission rates into account. According to our models, symbiotic had a significantly higher population growth rate than non-symbiotic plants, and endophyte prevalence was about 58%. Endophyte transmission rates were about 0.67 or 0.87, depending on the growth stage considered. In the presence of nutrient supplementation, population growth rates were still significantly higher for symbiotic than for non-symbiotic plants, but endophyte prevalence fell to 0%. At vertical transmission rates below 0.10–0.20, no symbiosis was observed. Our models showed that a positive benefit of the endophyte and vertical transmission rates of about 0.6 could lead to the coexistence of symbiotic and non-symbiotic *F*. *eskia* plants. The positive effect of the symbiont on host is not systematically associated with high transmission rates of the symbiont over short time scales, in particular following an environmental change.

## Introduction

Symbioses have been implicated in many of the major ecological and evolutionary innovations in the history of life [1]. For instance, the mitochondria of modern eukaryotes developed from an alpha-proteobacterium internalized by cells 1.45–2 billon years ago [2,3]. However, mitochondria and chloroplasts have become fixed across host populations and generations, whereas this is not the case for contemporary symbioses such as the fungus *Neotyphodium* in grasses (for a review [4]) or the bacterium *Wolbachia* in arthropods [5]. Consider the fungus from the genera Neotyphodium and Epichloë (Clavicipitaceae, Ascomycota) as an illustration. These vertically transmitted fungal endophytes are prevalent in cool-season grasses. They develop in the aerial tissues of the grass and are transmitted to the next generation of host plants via the seed. Fungal endophytes may confer several benefits on the host, increasing sexual reproduction rates, competitiveness and resistance to abiotic and biotic stresses, such as drought (see [6]) and herbivores (e.g. [7]). However, these vertically-transmitted fungal endophytes commonly display a mosaic of prevalence, ranging from 0 to 100%, in host populations [8–11]. Does this imply that contemporary symbioses are not sufficiently beneficial to their hosts to have become fixed? This hypothesis is intuitive and consistent with commonly understood principles of the mode of transmission and effects of symbiosis: exclusive vertical transmission would be expected to favor mutualism and to generate high frequencies of symbiosis in host populations [12,13]. Investigations of the interplay between symbiont transmission and effects in the generation of current prevalence patterns are required to improve our understanding of why some symbioses are fixed in host species, whereas others are not.

Theoretically, three principal mechanisms shape the overall pattern of variation of endophyte frequencies in a plant population: i) the outcome of symbiosis, in terms its benefits or harm to the host (e.g. [14]), ii) the mode of transmission (e.g. vertical and/or horizontal) and relative rates of transmission (e.g. [15]), and iii) the migration of symbiotic (S) and non-symbiotic (NS) organisms (i.e. via seeds) between neighboring populations [16]. Theoretical models suggest that imperfect transmission leads to the disappearance of the symbiont from the host population even when it is highly beneficial [17,18]. Recent empirical data are consistent with this hypothesis. Using a population modeling approach, Yule *et al*. [19] demonstrated that vertical transmission rates below a certain threshold could lead to symbiont extinction, even if the symbiont increased the net growth of *Agrostis hyemalis* populations. The proximal explanation for this is straightforward: the magnitude of the beneficial effect does not sufficiently compensate for the rate of symbiont loss. The distal explanation is much more complicated: why eliminate a beneficial symbiont, even if it is only slightly beneficial? The elimination of an advantageous endophyte over an evolutionary time scale is counterintuitive [12,13]. To our knowledge, this scenario of the elimination of an advantageous symbiont due to imperfect vertical transmission has never been demonstrated in a natural population, shaped by *evolutionary history*. It therefore remains to demonstrate whether and how it occurs in natural populations.

We considered two scenarios in which a positive effect of the endophyte was counteracted by weak transmission. These two scenarios did not result in the same probability of a fungal endophyte of grasses becoming fixed across host populations and generations. Under the first scenario, despite fluctuations over short time scales, there was a strong link between the transmission and effect of the symbiont. The positive effect was thus associated with weak transmission due to an episodic stress or disturbance. Evidence in favor of this “disturbance” hypothesis would be provided by a shift from high to low in the endophyte transmission rates. This shift should occur when environmental conditions are modified and in a population where the endophyte has a positive effect on the host. In the second scenario, the endophyte also increased host fitness, but its rate of transmission was low due to a trade-off between the benefit accrued and transmission. We called this scenario the “internal-limitation” hypothesis; it is an intrinsic characteristic of the symbiosis that precludes it transmission. As an example of this scenario, symbiosis may increase plant fitness by increasing vegetative growth rate, but high plant growth rates decrease the ability of the symbiont to colonize all the reproductive tillers of the plant (i.e. dilution effect; [20]). Evidence in favor of this scenario would be provided by a net positive effect of the symbiosis counteracted by a low transmission rate in a population with an intermediate steady-state endophyte prevalence. These scenarios lead to different evolutionary trajectories for the grass-endophyte symbiosis. The “disturbance” scenario enlarges the range of ecological conditions in which a symbiont can persist and be fixed across host generations. By contrast, in the “internal-limitation” scenario, the grass-endophyte symbiosis is unlikely to become fixed in host populations and generations with its current characteristics. Fixation is likely to occur only if a mutation arises that can disrupt the trade-off between symbiont advantage and transmission.

We analyzed the link between endophyte prevalence, effects and transmission in a natural population of an alpine grass, *Festuca eskia*, with intermediate levels of endophyte colonization. *F*. *eskia* harbors an asexual form of the endophytic fungus *Epichloë festucae*, and the proportion of symbiotic individuals in the population may range from 0 to 99%, depending on the location [10]. This plant species appears to be a simple and relevant model for investigations of the combination of mechanisms underlying the endophyte-grass system. Gene flow between populations is limited in this species [10], making it unnecessary to include migration processes in the model. We identified a population with an intermediate level of endophyte colonization (i.e. 50% of the plants symbiotic) over at least the last five years [10]. We used population modeling methods to estimate host-endophyte fitness. Given the connection between demography and fitness [21], population modeling appears to be an appropriate way of evaluating the outcome of symbiosis over the entire life cycle of the plant [19,22]. The use of this demographic approach in a wild population made it possible to assess the relative dynamics of symbiotic and non-symbiotic plants due to both environmental conditions and the life-history traits of the population. We focus here on the weighting of demographic parameters for determining the frequency of symbiotic and non-symbiotic plants, rather than on the predictive dimension in terms of population density. We addressed the following questions: 1) How are intermediate levels of endophyte colonization generated in a natural alpine grass population? 2) Are the transmission rates and effects on the host of the endophyte linked during ecological disturbances? 3) Which demographic traits can account for the differences between S and NS plants?

## Materials and Methods

### Study organisms

*Festuca eskia* Ram. (Poaceae) is a perennial grass endemic to the Pyrenees and Cantabrian Mountains from subalpine area (> 1500 m). It has been reported to be an outcrossing, wind-pollinated species [23]. It flowers from July to August, and the seeds mature from August to October. As in many perennial plants, it is not possible to estimate the age of the plant on the basis of above-ground morphological traits, and plants of *F*. *eskia* may potentially live *‘*forever’ [23]. *F*. *eskia* harbors an asexual form of the endophytic fungus *Epichloë festucae*. *Epichloë festucae* Leuchtmann, Schardl and Siegel (Ascomycota: Clavicipitaceae) is a fungal endophyte responsible for systemic, intercellular colonizations in cool-season grasses. The endophyte grows asymptomatically in the aerial tissues of the plant and can be transmitted vertically by the seed (asexual reproduction) or horizontally by ascospores (sexual reproduction) or conidiospores (asexual reproduction [24]). However, horizontal transmission has never been reported in *F*. *eskia*. We will use the term “endophytic symbiosis” to refer to the natural symbiotic relationship between *E*. *festucae* and *F*. *eskia*.

### Study site and population

Demographic data were collected for an *F*. *eskia* population in the Pyrenees (Guzet, N 42.78528° E 1.29806°, 1800 m above sea level). In June 2008, 100 symbiotic (S) and 100 non-symbiotic (NS) plants were randomly selected and labeled. This sample corresponds to 80% of the population. The endophytic status of each plant was determined with Phytoscreen Tissueprint immunoblot kits (Agrinostics Ltd, Inc., Watkinsville, GA, USA). The presence of *E*. *festucae* in plant tissues was confirmed for a subset of plants, by microscopy, as described by Hiatt *et al*. [25]. The endophytic fungus was identified as *E*. *festucae* by PCR, on the basis of β-tubulin sequences [10].

The environmental conditions were manipulated by adding nutrients to the soil, for half the population. Two nutrient resource treatments were established: natural and high-resource level (F+) treatments. Half the S plants and half the NS plants were assigned to each of these treatments. The plants assigned to the F+ treatment received 1.5 liters of nutrient solution (3.039 mg of N:P:K 19:2.6:10, for 1.5 L of snow melt water) every two weeks. This solution diffused steadily into the soil at the base of the tussock over a period of two weeks. The treatment was repeated, on the same plants in 2009 and 2010. Year-to-year variations in daily soil temperature and soil moisture content were recorded with a calibrated recorder (Hobo Station Logger H21 002 with Probe Smart S-TMA M002).

### Demographic traits

The population was examined over a period of three years (2008–2010, called years 1, 2 and 3) to estimate the plant size distribution, their survival and their fecundity. The size distribution of the *F*. *eskia* plant population was assessed by counting the total number of tillers on the 200 plants in October 2008 (year 1). In August 2009 (year 2), we determined the proportion of these 200 plants that were flowering. The number of seeds produced per plant was determined in October 2009 (year 2). Plant survival was monitored over the three-year period.

Sowing experiments were carried out to investigate seed dormancy and viability, seedling emergence and survival, and juvenile survival. In October 2008 (year 1), we harvested 50 mature seeds per plant from 20 S and 20 NS plants growing in natural conditions and 20 S and 20 NS plants subjected to the F+ treatment (total number of seeds = 4000). The seeds collected from the plants grown in natural conditions were sown in four plastic boxes (< 56 x 36 cm) filled with the local topsoil and left near by the natural population. The seeds collected from plants subjected to the F+ treatment were sown in four similar plastic boxes filled with a rich acid soil (92% loam, 8% clay; pH = 5.5; 700 ml/L N:P:K 15:20:24). From 2009 (year 2), seedlings were counted and removed at three-week intervals, during the period from snowmelt to snow cover, over a period of two years. Ten seedlings per mother plant were allowed to develop, for the assessment of juvenile survival and for determination of the number of tillers per juvenile, at three-week intervals. As no seedlings emerged in 2010 (year 3), the tetrazolium blue viability staining method was used to determine whether the remaining seeds were dead or dormant [26].

### Endophyte transmission rates

Vertical endophyte transmission rates in plants were estimated from adult plants to seeds, from seeds to seedlings and from seedlings to juvenile plants. Vertical transmission rates from adults to seed were established for at least five seeds per plant for 40 S plants (20 control, 20 F-treatment, n = 975 seeds). Endophytes were detected by staining with dilute aniline blue [27]. Rates of vertical transmission from seeds to seedlings and from seedlings to juveniles were estimated by determining the difference in endophyte frequency between seeds at the end of 2008 (year 1), seedlings at the end of 2009 (year 2) and juveniles at the end of 2010 (year 3, 200 seedling and 200 juveniles tested). Three tillers per juvenile were tested for endophytes with the Phytoscreen Tissueprint immunoblot kit. We checked that there was no horizontal transmission of the endophyte from S to NS plants at the end of the experiment.

### Matrix population models

**Fig 1:**
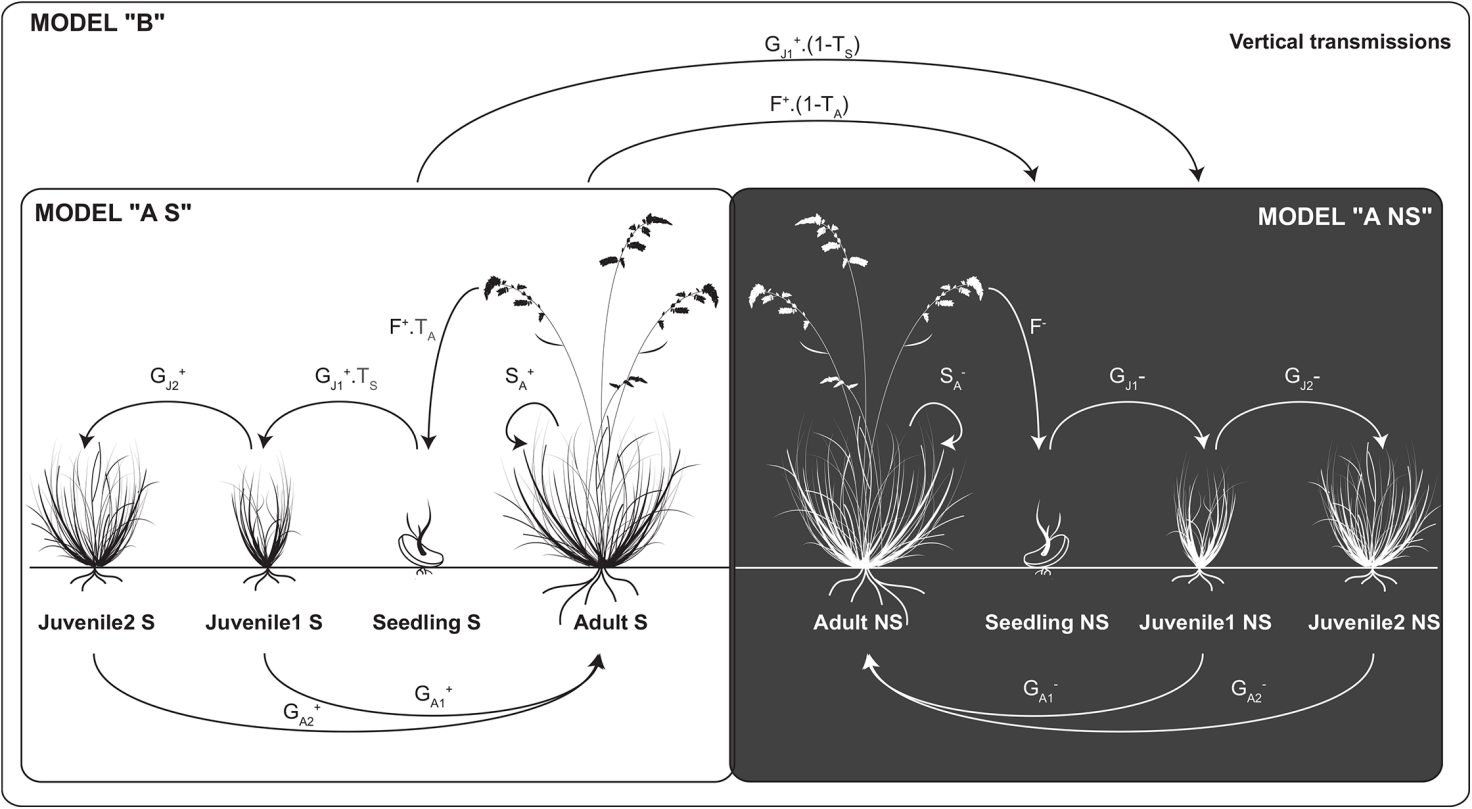
Diagram of the life cycle model for *Festuca eskia* populations including symbiotic and non-symbiotic plants. The model can be broken down into three submodels, according to the endophytic status of the plants present in the population: A^S^, A^NS^ and B. Globally, the model includes four symbiotic stages (Seedling S, Juvenile 1 S, Juvenile 2 S, Adult S), four non-symbiotic stages (Seedling NS, Juvenile 1 NS, Juvenile 2 NS and Adult NS), two probabilities for transitions between S and NS stages (Vertical transmissions: T_S_ from seedling S to Juvenile 1 NS; T_A_ from adult S to seed NS,), two fecundity arcs (F^+^ and F^-^), two survival probabilities (S_A_^+^ and S_A_^-^) and eight growth transitions (G_J1_^+^, G_J1_^-^, G_J2_^+^, G_J2_^-^, G_A1_^+^,G_A1_^-^,G_A2_^+^, G_A2_^-^).

Four stages in the life cycle of *F*. *eskia* were considered (Fig 1): i) seedlings, defined as young plants, less than one year old, ii) juveniles 1, defined as non-reproductive plants between one and two years old and with fewer than 30 tillers, iii) juveniles 2, defined as non-reproductive plants between two and three years old and with fewer than 30 tillers, and iv) adults, defined as reproductive plants that had reached the threshold of 30 tillers required for reproduction. This threshold of 30 tillers was established by determining the minimum size of all the reproductive plants at the Guzet site in 2008.

We developed two stage-structured matrix population models, with a time step of one year: i) model A, for which we developed two submodels, one consisting exclusively of S plants (model A^S^) and the other exclusively of NS plants (model A^NS^), and ii) model B, consisting of both S and NS plants, taking endophyte transmission rates into account (Fig 1). For matrix models A and B, we included six and 14 vital rates, respectively, calculated from our demographic traits and endophyte transmission rate censuses (Table 1, Fig 1). We parameterized six stage-structured projection matrices for the determination of NS, S and mixed *F*. *eskia* population growth rates, for the two levels of resources. The projection matrix of these models (S1 Fig) was linear and deterministic and was not density-dependent.

**Table 1:** Parameterization of elements in projection matrice.

	Definition	Equation
Matrix element		
G_J1_^+/-^	Probability of a seedling surviving and reaching the juvenile stage	s_1_
G_A1_^+/-^	Probability of a year-one juvenile surviving and reaching the adult stage	e_1_.s_2_
G_J2_^+/-^	Probability for a year-one juvenile surviving to the second year as a juvenile	(1-e_1_).s_2_
G_A2_^+/-^	Probability for a year-two juvenile surviving and reaching the adult stage	s_2_
S_A_^+/-^	Probability of an adult surviving	0.95
F^+/-^	Mean number of seeds produced by a plant that germinate to generate seedlings	(f_1_.P).e_0_
T_A_	Vertical transmission from adult to seed: probability of a seed produced by a symbiotic mother plant remaining infected	Σ(No. of symbiotic seed /No. of seed per symbiotic mother plant) / Σ mother plant
T_S_	Vertical transmission from seedling to juvenile: probability of an infected seedling remaining infected	Σ(No. of symbiotic juvenile /No. of symbiotic seedling) / Σ mother plant
Parameter		
s_1_	Probability of a seedling surviving	Σ(No. of seedlings alive /No. of seedlings emerged) / Σ mother plant
e_1_	Probability of a juvenile reaching 30 tillers	Σ(No. of juvenile1 of at least 30 tillers / No. of juvenile 1 sampled) / Σ mother plant
s_2_	Probability of a juvenile surviving	Σ(No. Juvenile alive /No. of juvenile followed) / Σ mother plant
f_1_	No. of seeds produced per flowering plant	Σ(No. of total seeds per plant) /(No. of flowering plant)
P	Probability of a plant flowering	Σ(No. of flowering plants) /(No. of total plants)
e_0_	Probability of seedling emergence from the seed	Σ(No. of emerged seedlings /No. of seeds sown) / Σ mother plant

These models were developed under the following assumptions: 1) A fixed adult survival rate (S_A_) of 0.95, and 2) The loss of the fungal endophyte from an S plant results in demographic behavior equivalent to that of an NS plant (model B). We monitored the population for three full years under harsh alpine conditions, but we observed no dead plants among the established individuals. As a consequence, the adult survival rate was fixed at 0.95, and simulations were performed with a range of values (from 0.5 to 0.99). Based on our expert knowledge of this system, there was no reason to attribute different survival rate to S and NS plants. Indeed, stone avalanches constitute the principal risk of death for adult *F*. *eskia* plants, and endophytes were not expected to have any effect on the probability of a plant being carried away by such an avalanche.

### Simulation of population growth and disturbance analysis

For each of the six transition matrices, the asymptotic population growth rate (λ) was calculated by Monte Carlo simulation (50 time steps, 1000 trajectories), with ULM (Unified Life Model) software [28,29]. We used Monte Carlo simulation, to include uncertainty due to the well known strong endophyte*plant genotype effect in the population growth rates (see [4]).

For model A, we performed prospective and retrospective analyses, to explore the effect on population growth rate of differences in vital rates [30]. Prospective analyses (i.e elasticity analysis) consider the consequences of potential future changes in vital rates for population growth [30]. The retrospective analyses (i.e. fixed-effect life table reponse experiment LTRE) were designed to quantify the contribution of each vital rate to the effect of endophytic status and resource level on population growth rate [21,30]. The matrix of the A^NS^ model under natural conditions was used as the reference matrix in the separate LTRE analyses. Model B generated an additional output: the proportion of S plants in the population, corresponding to the frequency of the fungal endophyte in the population. We used model B to simulate changes in endophyte frequencies and in λ in the *F*. *eskia* population, as a function of resource level and vertical transmission rates (T_S_ and T_A_). These simulations were carried out by fixing one of the two transmission rates at the observed values, and by varying the other transmission rate from 0 to 1.

### Statistical analyses, availability of code and data

We used a generalized linear model with a logit link function to check for differences in model parameters between endophyte statuses and resource levels, assuming that the errors were binomially distributed. The model considered three factors as fixed effects: endophytic status, edaphic resource level and their interaction. Significance was assessed in a χ^2^ analysis of deviance. Differences in mean population growth rates were analyzed in *t*-tests. All analyses were conducted with R software [31] or ULM (Unified Life Model) software [28,29]. The elasticity analysis was performed with ULM software. The fixed-effect life table response experiment (LTRE—a retrospective approach) was conducted with R software version 3.1.2 [31] and the popbio package [32], using Caswell’s equations. The code and data underpinning this paper is available at github.com/AnaisGibert/DemographicModel. Archival copies are also included in Supplementary material (S1 Text).

### Ethics statement

This study was carried out on land managed by the Altiservice company, at the Guzet ski resort. The manager of the site, Mr Akim Boufaïd, and the president of the local pastoral group using this site in the summer, Mr Marcel Fort, gave permission for the study to be conducted at this site. We confirm that this field study involved no endangered or protected species.

## Results

### Life cycle of *F*.*eskia*

**Fig 2:**
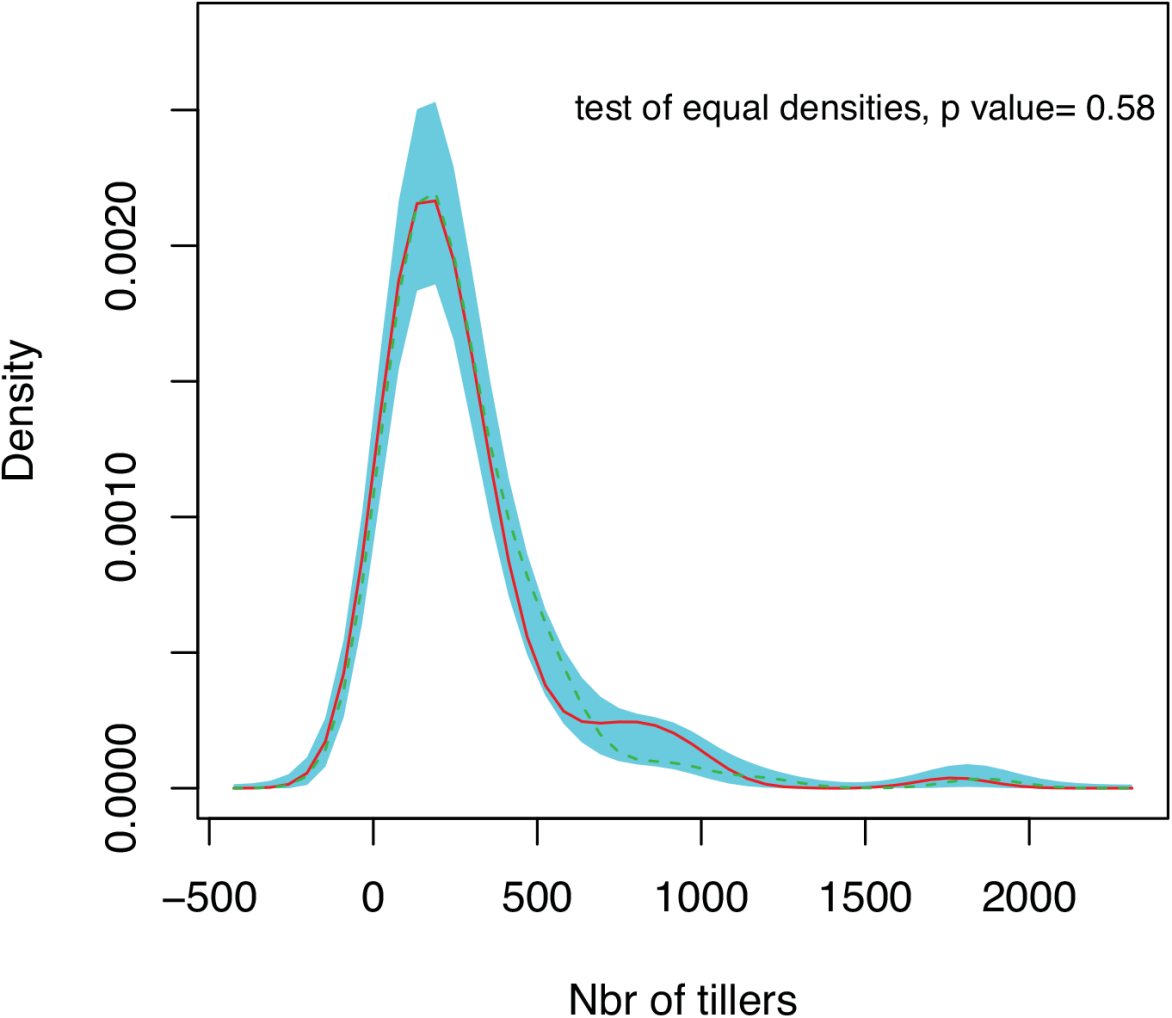
Size distribution of *Festuca eskia* plants at the Guzet site (France), as a function of the presence (red line) or absence (green line) of the fungal endophyte *Epichloe festucae*. CI±95% is in turquoise.

The studied *F*.*eskia* population contains fewer than 250 plants, most with 30 to 1200 tillers, with a few plants bearing up to 3000 tillers. The size distributions of S and NS plants were similar: we compared density in S and NS both graphically and formally in a permutation test of equality and show no differences (Fig 2, see kernel density comparison p value = 0.58). In adult plants, resource allocation to reproduction (RA, number of seeds / number of vegetative tillers) was not dependent on plant size (results of non parametric ANCOVA p value = 0.87), demonstrating it was the most parsimonious solution to consider the adult plant stage as a single category in our model. The 5% of seeds that did not germinate after one year were all dead, demonstrating an absence of seed dormancy or of a seed bank for *F*. *eskia*. Finally, the 25 plants initially identified as NS remained endophyte-free throughout the entire three-year study, suggesting no endophyte horizontal transmission.

### Environmental variations

Year-to-year variations in daily soil temperature and soil moisture content were not significant during our study (mean of 5.08 ±0.06°C in year 2 vs. 5.31 ±0.06°C in year 3 and 0.17 ±0.001 m^3^ of water. m^-3^ of soil in year 2 vs. 0.20 ± 0.0006 m^3^ of water. m^-3^ of soil in year 3; mean ± SEM; p >0.05). It was therefore possible to use assessments of the performance of host-stage classes to separate the effects of host development from those of temporal variation, because there was no significant year-to-year variation in soil moisture content or air humidity and temperature.

### Effects of symbiosis and resource level on plant demographic traits

The probability of seedling emergence from the seed (e_0_) was the demographic trait found to vary significantly as a function of the interaction between plant endophytic status and resource level (Table 2). In natural conditions, the probability of seedling emergence was 16 percentage points (pp) lower in NS compared to S plants. By contrast, the F+ treatment resulted in the same probability of seedling emergence between S and NS plants. The probability of a seedling surviving (s_1_) was found to vary significantly as a function of plant endophytic status or resource level (Table 2). The probability of a seedling surviving was 14pp higher under F+ treatment than for the natural conditions, and 6pp higher in S than in NS plants. The probability of a juvenile surviving (s_2_) was found to vary significantly as a function of resource level. The probability of a juvenile surviving was 29pp higher under F+ treatment than for the natural conditions.

**Table 2:** Mean and results of deviance analysis for the parameters of the models of *Festuca eskia* population dynamics. The values shown are means ±SEM.

	Means				*P values*		
Edaphic resource level	Control		F+ treatment		Endophytic status	Resource level	Interaction
Endophytic status	NS	S	NS	S			
Parameter^a^							
s_1_	0.79 ±0.036	0.85 ±0.028	0.95 ±0.012	0.97 ±0.009	**0.041**	**<5.22e-10**	0.882
s_2_	0.63 ±0.095	0.69 ±0.078	0.91 ±0.042	0.99 ±0.007	0.155	**<2.22e-06**	0.169
e_1_	0.05 ±0.019	0.08 ±0.009	0.11 ±0.011	0.09 ±0.018	0.473	0.051	0.205
P	0.59 ±0.058	0.66 ±0.054	0.72 ±0.091	0.80 ±0.082	0.521	0.319	0.975
f_1_	123 ±38	125 ±40	77 ±52	184 ±70	0.336	0.831	0.299
e_0_	0.31 ±0.047	0.48 ±0.020	0.45 ±0.040	0.42 ±0.023	**0.019**	0.291	**0.005**
T_A_	—	0.63 ±0.030	—	0.52 ±0.021	—	0.331	—

^a^ See Table 1 for parameter definitions; *P-values*<0.05 are shown in bold.

S: symbiotic; NS: non-symbiotic.

Vertical transmission rates in *F*. *eskia* varied with life-cycle stage (from adults to seeds or from seedlings to juveniles, *p* < 0.005) and resource levels (*p* < 0.001), whereas the interaction between these two factors was not significant (*p* = 0.7988). The overall rate of vertical transmission was higher under natural conditions than for the F+ treatment (natural conditions: 0.75 ±0.01 vs. F+ treatment: 0.67 ±0.01, *p* < 0.001, Table 3). The endophytes were more likely to be lost between the adult and seed stages (T_A_: 0.58 ±0.019; mean ± SEM; total scored: n = 975 seeds), or between the seedling and juvenile stages (T_S_: 0.85 ±0.021, total scored: n = 200 juveniles), than between the seed and seedling stages (0.97 ±0.010; total scored: n = 200 seedlings). The transmission rates between the adult and seed stages (T_A_) and between the seedling and juvenile stages (T_S_) were found to vary but not significantly as a function of resource level (Table 2); T_A_ decreased of 11pp and T_S_ of 5pp from natural condition to F+ treatment.

**Table 3:** Summary of endophyte effect, transmission rates and prevalence values in *F*.*eskia* under two resource levels. Growth rates were obtained from the model A, the overall endophyte transmission were measured in the field, the endophyte prevalence were returned by the model B and the threshold in transmission below which the symbiont frequency in the population was zero were obtained by simulations. T_A_ transmission rate from adult to seed, T_S_ transmission rate from seedling to juvenile.

	Population growth rates (λ)		Overall Endophyte transmission	Endophyte prevalence (%)	Thershold below which the symbiont frequency in the population was zero	
	NS plants	S plants			T_A_	T_S_
Control condition	1.97 ±0.21	2.34 ±0.26	0.75 ±0.01	58%	0.1	0.2
F+treatment	2.45 ±0.17	3.17 ±0.23	0.67 ±0.01	0%	0.6	0.9

### Effects of symbiosis and resource level on population growth rates

The growth rates of symbiotic and non-symbiotic populations were significantly different under natural conditions (Table 4: NS: 1.18±0.025 vs. S: 1.44± 0.051, *p* < 2.2e-16). Similarly, S plants had a significantly higher rate of population growth than NS plants under F+ treatment (Table 4: NS-F: 1.65± 0.073 vs. S-F: 1.94± 0.089; *p* < 2.2e-16). High edaphic resource levels had similar effects on S and NS plants. The growth rates of S and NS populations increased significantly with edaphic resource level (around 25% of increase, *p* < 2.2e-16). According to model B, consisting of both S and NS plants and taking endophyte transmission rates into account (Fig 1), the prevalence of endophytes in the population is about 58% under natural conditions, whereas it falls to 0% for the F+ treatment (Table 3). For vertical transmission rates fixed at 1 (i.e. perfect vertical transmission), endophyte prevalence reaches 100% for both conditions (S2 Fig).

**Table 4:** Transition, elasticity matrices, reproductive values and stable distribution across stages of *Festuca eskia* as a function of endophyte status and edaphic resource level. The transition matrices for each set of conditions contain the life-cycle stage and the probabilities. The values shown are means ± SEM. F+treatment: addition of fertilizer. Λ: asymptotic population growth rate calculated by Monte Carlo simulation.

	Transition matrix				Elasticity matrix				Reproductive value	Stable distribution
	Seedling	Juvenile1	Juvenile2	Adult	Seedling	Juvenile1	Juvenile2	Adult		
Non-Symbiotic, control condition (λ = 1.97 ±0.21)										
Seedling	_	_	_	(73 ±27) x (0.31 ±0.216)	0	0	0	0.21	3	62
Juvenile1	0.79 ±0.166	_	_	_	0.21	0	0	0	8	25
Juvenile2	_	0.60 ±0.026	_	_	0	0.18	0	0	21	8
Adult	_	0.032 ±0.049	0.63 ±0.437	0.95	0	0.03	0.18	0.19	68	5
Symbiotic, control condition (λ = 2.34 ±0.26)										
Seedling	_	_	_	(83 ±26) x (0.48 ±0.089)	0	0	0	0.23	3	66
Juvenile1	0.85 ±0.16	_	_	_	0.23	0	0	0	7	24
Juvenile2	_	0.58 ±0.039	_	_	0	0.17	0	0	20	6
Adult	_	0.055 ±0.045	0.69 ±0.355	0.95	0	0.05	0.17	0.15	70	4
Non-Symbiotic, F+treatment (λ = 2.45 ±0.17)										
Seedling	_	_	_	(55 ±17) x (0.45 ±0.145)	0	0	0	0.23	4	62
Juvenile1	0.95 ±0.144	_	_	_	0.23	0	0	0	10	24
Juvenile2	_	0.81 ±0.045	_	_	0	0.17	0	0	23	8
Adult	_	0.09 ±0.044	0.90 ±0.166	0.95	0	0.05	0.18	0.14	63	6
Symbiotic, F+treatment (λ = 3.17 ±0.23)										
Seedling	_	_	_	(149 ±28) x (0.42 ±0.1)	0	0	0	0.24	3	69
Juvenile1	0.97 ±0.031	_	_	_	0.24	0	0	0	8	21
Juvenile2	_	0.90 ±0.079	_	_	0	0.18	0	0	21	6
Adult	_	0.087 ±0.077	0.99 ±0.033	0.95	0	0.06	0.18	0.1	68	4

### Prospective (elasticity) and retrospective (LTRE) analyses

**Fig 3:**
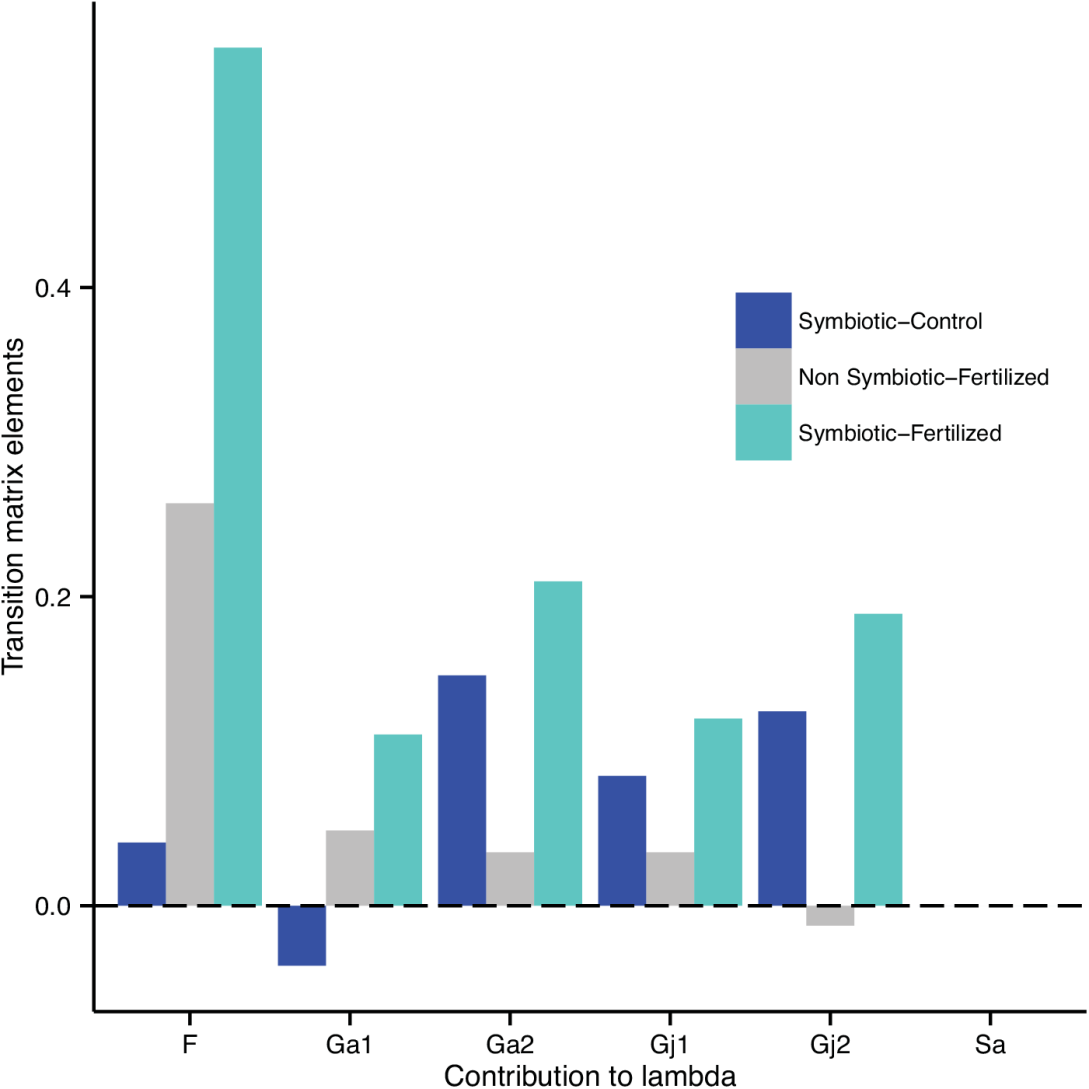
Contributions of matrix elements to the variability of λ for *Festuca eskia* populations, as a function of endophytic status and edaphic resource level.

The probability for a year-two juvenile surviving and reaching the adult stage (G_A2_) and the probability for a year-one juvenile surviving to the second year as a juvenile (G_J2_) had the lowest elasticity for all matrices: these parameters accounted for 0% of the summed elasticity values of all matrix elements (Table 4). By contrast, elasticity values were highest for the probability of a seedling surviving and reaching the juvenile stage (G_J1_), for the mean number of seeds produced per plant (F), and for the probability for a year-one juvenile surviving and reaching the adult stage (G_A1_), each of which accounted for 23 to 29% of the summed elasticity values for all matrix elements (Table 4). LTRE analysis showed that the mean number of seeds produced per plant (F) was the major contributor to the differences in lambda between NS and S populations for the F+ treatment (Fig 3). All the other parameters contributed to this difference, but to lesser extent. The mean number of seeds produced per plant (F) also made a contribution to the difference in lambda between S and NS populations under natural conditions.

### Simulation of population growth rate and endophyte frequencies as a function of endophyte transmission rates

**Fig 4:**
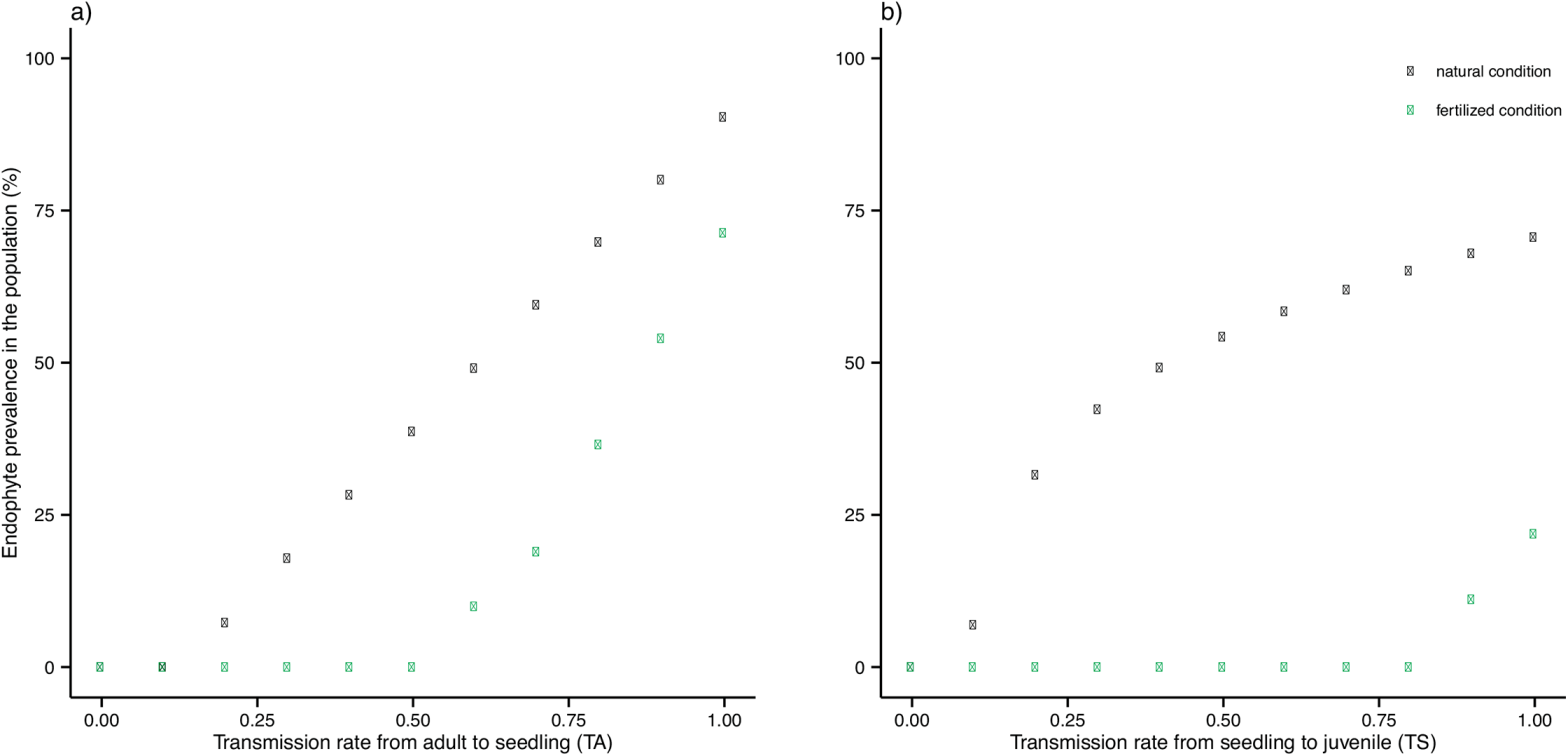
Endophyte prevalence (%) in host populations, as a function of edaphic resource level and variations in (a) vertical transmission rate from symbiotic adult to non-symbiotic seed (TA) with TS = 1, and (b) vertical transmission rate from symbiotic seedling to non-symbiotic juvenile 1 (TS) with TA = 1. Natural: black symbols; F-treatment: green symbols. See Fig 1B for life cycle and Table 2 for the values of the parameters used in the model.

Population growth rates varied with vertical endophyte transmission rate from adult to seed, and from seedling to juvenile (S2 Fig). An increase in vertical transmission rates led to higher population growth rates. Variations of vertical transmission rates (TA and TS) led to changes in endophyte prevalence (Fig 4). The threshold beyond which vertical transmission was not compensated by the effect of the endophyte on growth rate—i.e. the threshold at which endophyte prevalence decreased to zero—was around 0.1–0.2 under natural condition (0.2 for TA and 0.1 for TS, Table 3). This threshold increased under F+ conditions (0.6 for TA, and 0.9 for TS, Table 3). Variation of the rate of vertical transmission from adult to seed (TA) led to a greater variation of endophyte prevalence (ranging from 0 to 91% of S plants under natural conditions, and 0 to 71% for the F+ treatment Fig 4B) than variation of the rate of vertical transmission from seedling to juvenile (TS, ranging from 0 to 70% of S plants under natural conditions, and 0 to 21% for the F+ treatment Fig 4A).

## Discussion

To our knowledge, this study is the first to describe the mechanistic links between endophyte prevalence, effects on host fitness and transmission in a natural grass population. Our findings suggest that symbiotic and non-symbiotic plants can coexist in a stable manner within grass populations, due to the combination of a positive effect of the endophyte on host fitness, and imperfect vertical transmission. We also demonstrated that changes to nutrient resources dissociated the transmission rate of the endophyte from its effects, leading to the disappearance of the endophyte from the host population.

These findings are clearly consistent with the “disturbance” hypothesis. The transmission and effects of the symbiont can be dissociated over short time scales in the presence of a disturbance (a change in soil nutrient levels in this study). However, they are also consistent with the “internal-limitation” hypothesis. Under this hypothesis, a positive effect of the symbiosis would be counteracted by weak transmission, even in the lack of an environmental disturbance, due to a trade-off between symbiont benefit and transmission. Our results showed a positive effect of the endophyte on population growth rate, in the presence of transmission rates of about 0.63 to 0.87. A transmission threshold of 0.1–0.2 was calculated, below which the symbiont frequency in the population was zero. The transmission rates observed in this study are consistent with those reported in the literature (about 0.92 for *Epichloë* species and 0.75 for *Neotyphodium* species, see [33]). However, our result raises questions about how “weak” transmission should be defined. Exclusively perfect vertical transmission is known to be rare in symbiotic systems. With the exception of mitochondria and chloroplasts, all vertically transmitted symbionts display some plasticity in their rates of vertical transmission (see [34]). The use of terms such as “imperfect transmission” in endophyte-grass symbiosis assumes that perfect transmission is the baseline condition, but quantitative evaluations of transmission rates have shown that these rates range from 0 to 1 [33,35]. Further studies are clearly required to establish the physiological link between plant growth, fecundity and endophyte transmission.

### How are intermediate levels of endophyte colonization achieved in a natural alpine grass population?

Our findings identify a positive effect of the endophyte and imperfect vertical transmission as factors accounting for the stable coexistence of symbiotic and non-symbiotic plants of *F*. *eskia*. Indeed, in our model, the positive effect of the endophyte on symbiotic plants was enough to account for the preponderance of endophytes in grass populations. However, this positive effect was counteracted by intermediate rates of vertical transmission (i.e. 0.63 to 0.87). Our results are consistent with the predictions of several theoretical models. Those of Ravel *et al*. [36] and Gundel *et al*. [18] predict the persistence of non-symbiotic grasses in a population, under an assumption of non-propagation of the endophyte.

We demonstrated that symbiotic and non-symbiotic plants could coexist in a stable manner within grass populations. Intermediate endophyte prevalences are the norm, rather than the exception, in grass species [9,33], but only a few empirical studies have focused on populations with intermediate symbiotic frequencies. Instead, most studies have focused on individuals from populations with high symbiotic frequencies. For example, Yuel *et al*. [19] studied native grasses for which 97% of the seeds contained the symbiotic endophyte. Davitt *et al*. reported an endophyte prevalence of 96% and Kannadan and Rudgers [37] reported an endophyte prevalence of about 74 to 100%. Populations with intermediate frequencies have generally been considered not to be in equilibrium and to display transient dynamics [18], due to environmental fluctuations or seed immigration [16,38]. Our work thus clarifies the status of populations with intermediate symbiotic frequencies, and provides a rationale for further studies of the specific trajectories underlying the establishment of intermediate frequencies.

### Are these two mechanisms linked in conditions of ecological disturbance?

Our findings indicate that the two mechanisms identified act independently following a change in soil resource level. When fertilizer was applied (F+), the fungal endophyte enhanced *F*. *eskia* fitness, but vertical transmission rates were slightly lower than in the absence of fertilizer, resulting in a frequency of *E*. *festucae* of 0% in the population. The apparent paradox between the enhancement of host fitness by the endophyte and the decrease in endophyte transmission observed in our study indicates that these two mechanisms are not linked by a monotonous relationship over the ecological time scale. It is therefore not possible to estimate one mechanism directly from the other. From an ecological perspective, our results are consistent with those of Gibert *et al*. [35], showing, for several *F*. *eskia* populations with 11 to 90% S plants, an apparent discrepancy between the effect of the endophyte on the host and the rate of vertical endophyte transmission.

### Which demographic traits can explain the difference between S and NS plants?

The prospective analysis (elasticity) identified three demographic parameters as particularly likely to affect population growth rate in *Festuca eskia*: the probability of a seedling surviving to the juvenile stage (G_J1_), the probability for a year-one juvenile surviving and reaching the adult stage (G_A1_) and the mean number of seeds produced per plant (F). However, only a component of F, e_0_ the probability of seedling emergence from the seed, explained the observed differences in population growth rates between NS and S plants in both sets of conditions. This finding is quite logical for a symbiosis in which seeds are the vector for the transmission of the symbiont across host generations. Inconsistent effects of endophyte on plant germination rates have been reported in the literature (see [39]). It would therefore be of interest to investigate the beneficial effects of the endophyte (resistance to drought stress, etc.) through this demographic parameter.

Theoretical and empirical studies have predicted the existence of a threshold vertical transmission rate, below which the positive effect of the endophyte cannot compensate for the rate of symbiont loss [17–19]. Here, we document the occurrence and magnitude of such a transmission threshold in a natural system: at vertical transmission rates below 0.1–0.2 under natural condition–the precise threshold depending on the life-cycle stage considered—the frequency of symbionts in the population was zero in our study. This result is consistent with both the theoretical results of Ravel *et al*. [17], and the empirical results of Yule *et al*. [19], who predicted that symbiotic grasses could persist in the population, provided that transmission rates exceeded 0.1. Our results also demonstrated that this threshold was higher under F+ treatment than under control conditions, suggesting that it could change across environmental gradient. Our results also indicate that vertical transmission from adult to seed (TA) is more critical for the persistence of symbiosis than vertical transmission from seedling to juvenile (TS) in a *F*.*eskia* population. In our study, the vertical transmission threshold beyond which symbiosis persisted in the population was higher for TA (i.e. 20%) than for TS (i.e. 10%) under natural conditions. This result is consistent with previous studies reporting the tiller-seed transition (TA) as the transition during which symbiosis is most frequently lost in grass-endophyte systems (see [33]). Yet, the relative importance of the vertical transmission parameters appears to be dependent on environmental conditions. Indeed, in our study the vertical transmission threshold beyond which symbiosis persisted in the population was higher for TS (i.e. 20%) than for TA (i.e. 10%) under fertilized conditions, suggesting that the different vertical transmission parameters have to be studied simultaneity.

### Model validation

The assumption of a fixed survival rate of 0.95 in adult, in addition to the use of a sowing experiment, resulted in an overestimation of population growth rates in our study. The absolute values of lambda were > 1 (i.e. expanding population). However, the ranking of population growth rates between symbiotic, non-symbiotic, natural and fertilized conditions is more important than the absolute value of growth rate in this study. And this ranking was not affected by the choice of an arbitrary value for survival (here 0.95, but simulations were performed with a range of values, from 0.5 to 0.99, result not shown). Finally, the endophyte prevalence values estimated by our model are consistent with observation from the *Festuca eskia* population in Guzet,

## Conclusion

In conclusion, we have demonstrated that the combination of a small positive effect of the endophyte on host fitness and imperfect vertical transmission can generate an intermediate steady-state prevalence of an endophyte in a natural population. Our findings also reveal that the link between the effect of a symbiont and its transmission may not apply over the ecological timescale, particularly in the presence of ecological disturbances. Finally, our results highlight the need for additional empirical studies of the physiological processes connecting plant growth, reproduction and endophyte transmission.

## Supporting Information

S1 Fig The projection matrices of two stage-structured population models, with a time step of one year. Model A consists either exclusively of symbiotic plants (model A^S^) or of non-symbiotic plants (model A^NS^). Model B consists of both S and NS plants and takes endophyte transmission rates into account. See Fig 1B for life cycle and Table 2 for the values of the parameters used in the model.
(EPS)

S2 Fig Relationship between a) Endophyte prevalence (%) in host populations, and b) growth population rate (lambda) as a function of both the vertical transmission rate from symbiotic adult to non-symbiotic seed (TA) and the vertical transmission rate from symbiotic seedling to non-symbiotic juvenile 1 (TS) ranging from 0 to 1. See Fig 1B for life cycle and Table 2 for the values of the parameters used in the model. Data reported only for natural conditions.
(EPS)

S1 Text The data (parameter, and simulations) used in this paper. (ZIP)
